# Acceptability, Feasibility, and Cost of Telemedicine for Nonacute Headaches: A Randomized Study Comparing Video and Traditional Consultations

**DOI:** 10.2196/jmir.5221

**Published:** 2016-05-30

**Authors:** Kai Ivar Müller, Karl Bjørnar Alstadhaug, Svein Ivar Bekkelund

**Affiliations:** ^1^ Department of Neurology University Hospital of North Norway Tromsø Norway; ^2^ Department of Clinical Medicine UiT – The Arctic University of Norway Tromsø Norway; ^3^ Department of Neurology Nordland Hospital Trust Bodø Norway

**Keywords:** headache, management, consultation, telemedicine, burden, cost, feasibility, rural, randomization

## Abstract

**Background:**

The feasibility of telemedicine in diagnosing and treating nonacute headaches, such as primary headaches (migraine and tension-type) and medication-overuse headaches has not been previously investigated. By eliminating the need of travel to specialists, telemedicine may offer significant time and money savings.

**Objectives:**

Our objective was to estimate the acceptance of telemedicine and investigate the feasibility and cost savings of telemedicine consultations in diagnosing and treating nonacute headaches.

**Methods:**

From September 2012 to March 2015, nonacute headache patients from Northern Norway who were referred to neurologists through an electronic application system were consecutively screened and randomized to participate in either telemedicine or traditional specialist visits. All patients were consulted by two neurologists at the neurological department in Tromsø University Hospital. Feasibility outcomes were compared between telemedicine and traditional groups. Baseline characteristics and costs were then compared between rural and urban patients. Travel costs were calculated by using the probabilistic method of the Norwegian traveling agency: the cheapest means of public transport for each study participant. Loss of pay was calculated based on the Norwegian full-time employee’s average salary: < 3.5 hours=a half day’s salary, > 3.5 hours spent on travel and consultation=one day’s salary. Distance and time spent on travel were estimated by using Google Maps.

**Results:**

Of 557 headache patients screened, 479 were found eligible and 402 accepted telemedicine participation (83.9%, 402/479) and were included in the final analyses. Of these, 202 received traditional specialist consultations and 200 received telemedicine. All patients in the telemedicine group were satisfied with the video quality, and 198 (99%, 198/200) were satisfied with the sound quality. The baseline characteristics as well as headache diagnostics and follow-up appointments, and the investigation, advice, and prescription practices were not statistically different between the two randomized groups. In addition, telemedicine consultations were shorter than traditional visits (38.8 vs 43.7 min, *P*<.001).

The travel cost per rural individual (292/402, 73%) was €249, and estimated lost income was €234 per visit. The travel cost in the urban area (110/402, 27%) was €6, and estimated lost income was €117 per visit. The median traveling distance for rural patients was 526 km (range 1892 km), and the median traveling time was 7.8 hours (range 27.3 hours). Rural patients had a longer waiting time than urban patients (64 vs 47 days, *P*=.001), and fewer women were referred from rural areas (*P*=.04). Rural women reported higher pain scores than urban women (*P*=.005).

**Conclusion:**

Our study shows that telemedicine is an accepted, feasible, time-saving, and cost-saving alternative to traditional specialist consultations for nonacute headaches.

**Trial Registration:**

Clinicaltrials.gov NCT02270177; http://clinicaltrials.gov/ct2/show/NCT02270177 (Archived by WebCite at http://www.webcitation.org/6hmoHGo9Q)

## Introduction

Nonacute headaches are among the most frequent disorders in humans [1,2]. The global burden of these headaches (eg, migraine, tension-type, and medication overuse headaches (MOH)) account for the most common neurological cause of disability-adjusted life years and years lived with disability (45.1% and 69.3% of the total in 2013, respectively) [2,3]. Moreover, nonacute headaches represent a frequent cause of referrals to neurologic outpatient clinics and are considered to be a cause of the third largest neurological health cost in Europe, estimated annually at €43,514 million in 2010 [4,5].

Patients with nonacute conditions need a referral to be accepted to a specialist consultation according to Norwegian health laws. Nonacute headaches usually occur for at least four weeks without any clinical or radiological signs of structural intracranial pathology [6]. Patients in our study are diagnosed according to the second version of the International Classification of Headache Disorders 2 (ICHD-2) [7]. The most frequent primary headaches are migraines and tension-type headaches, while an important cause of chronic headache is MOHs. These also constitute the majority of nonacute headaches.

Northern Norway’s physical geography is extensive with many sparsely populated areas. Access to specialist health care becomes cumbersome, expensive, and time consuming for many patients because of variable weather conditions. Efforts to facilitate easier access to specialists for headache patients are furthermore obstructed by tight health budgets. Accordingly, the rules and regulations surrounding the practice of telemedicine in Norway are addressed in a government circular letter of 2001 [8]. In general, this document states that consultations, diagnostics, treatments, and safety issues with telemedicine are governed by the same principles as traditional face-to-face consultations.

Telemedicine may help reduce the burden for neurological patients who live in rural or underserved areas [9,10], and it is considered equal in quality to traditional visits among different medical professions [11]. Guidelines for telemedicine in a second opinion for headaches have been suggested [12], but the feasibility of telemedicine being used as a tool for outpatient headache specialist consultations has only been addressed in case series [13-16].

A 2013 review of telemedicine interventions for somatic diseases found that 23% of papers (7/31) documented effectiveness or cost-effectiveness and 42% (13/31) showed promising results [17]. However, the evidence for health costs and patient acceptability in telemedicine was nonconclusive according to a 2013 Cochrane review [16]. Lack of such evidence hampers implementation of new information and communication technologies at the expense of patients’ needs [18].

The aim of this study was to evaluate patient acceptance. We also sought to investigate the feasibility of using telemedicine in order to hinder access barriers by being independent of the patient’s and headache specialist’s location. Additionally, our study also estimated cost savings by consulting headache patients via telemedicine in the spectrum of headaches referred from general practice.

## Methods

### Study Design

Feasibility and economic evaluations are part of an ongoing open-labeled noninferiority randomized clinical trial (Clinicaltrial.gov id. NCT02270177). In this trial, specialist telemedicine visits versus traditional specialist visits for headache sufferers were compared.

### Study Population

The study was conducted in the neurological outpatient clinic at the University Hospital of Northern Norway in Tromsø city. This facility serves 190,726 inhabitants in Troms county and upper Nordland county, which are distributed over an area of 25,877 km^2^ [19]. People living in areas further toward north, as in Finnmark and Svalbard, are also served by the same clinic. Finnmark has 75,605 inhabitants spread over a land area of 48,618 km^2^, and Svalbard has a Norwegian population of 2180 inhabitants spread over an area of 61,022 km^2^ [19]. In general, individuals from Finnmark and Svalbard travel by plane. Individuals from Troms and Nordland travel by car, bus, or boat.

We screened all patients who were referred to specialists for headaches from September 30, 2012 to March 30, 2015. The inclusion criteria were as follows:

1. Females and males aged ≥16 and ≤65 years

2. Referred to a neurologist for headache diagnostic clarification and/or treatment

3. Lack of abnormal findings on either clinical neurological examination, reported by the referring doctor, or by imaging of the brain suggestive of a secondary cause

4. Waiting time ≤4 months from date of the referral letter

5. Speaking Norwegian language.

To prevent working with patients already diagnosed with headaches, those who had been evaluated by a neurologist for 2 years before referral were excluded.

The legal age of consent in Norway is 16 years. To reduce the risk of including patients with secondary headaches and to recruit from a working population, patients above 65 years of age were excluded. In general, eligibility criteria were set to prevent working with participants unsuitable for telemedicine. Therefore, referrals of acute and secondary headaches, or evidence of such, were excluded. For recruitment purposes and to prevent outdated information in the referral letters, the waiting time for a consultation was set to 4 months or less. Speaking the Norwegian language was considered a necessity to ensure proper and accurate communication.

### Recruitment and Randomization

All eligible patients were identified through an electronic application system present in the distributed information system and patient system for hospitals from DIPS AS (DIPS) at the hospital. These individuals received an information letter and were interviewed by a study coordinator for eligibility criteria, telemedicine acceptance, and study participation. Individuals who did not meet the criteria or did not want to participate were transferred back to the traditional consultation setup. Next, candidates were called for final information and participation. Volunteers then received a consent form, a questionnaire, and a summoning letter. A study coordinator consecutively scheduled the participants for consultation, and they all met at the neurological outpatient department at Tromsø University Hospital.

**Figure 1:**
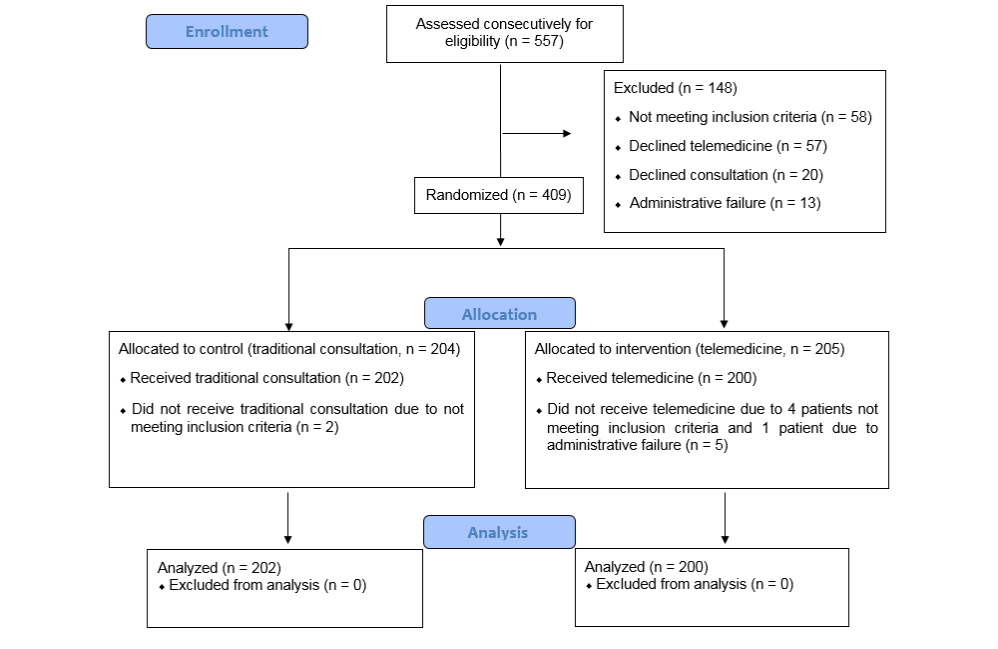
Flow of participants through the study.

A study nurse subsequently called the randomization office at the hospital and followed each patient to the consultation. The consultations took place between 9 am and 3 pm. Patients were block randomized by using Microsoft Access. The block sizes varied among four, six, and eight, and stratification was made on each neurologist. Furthermore, patient preparation time and consultation time were recorded. A study flow chart is given in Figure 1.

### Equipment

Video consultations were performed by using a videoconference system (Cisco C40 integrator package, Cisco C40 Integrator Multisite, Cisco Precision HD 1080p 12xcamera, NEC X551s 55” LED monitor, Audio-Technica ceiling microphones and JBL LSR2325P active speakers, Integrator Package C40 dual display option, and Cisco touch-control device for C Series) that was installed in one office. The neurologist consulted the patients from two other offices via a Cisco EX60 unit with an InTouch panel. Moreover, face-to-face consultations were performed in a traditional manner from the same offices.

### Outcome Variables and Measurements

All data, including background variables, were recorded by structured interviews using an administrative protocol. Nonacute headaches were categorized according to the diagnosis and frequency into three groups: <7 days, 7-15, and ≥15 days per month within the last 3 months before consultation. Headaches occurring for ≥15 days per month for more than 3 months were defined as chronic while those occurring for <7 or 7-14 days were termed episodic [7].

A horizontal visual analog scale (VAS) ranging from 0 to 10, which has demonstrated validity and reliability for many pain conditions including headaches, has been in use both in research and clinical settings [20]. This method was used to measure headache intensity (0=no pain and 10=worst possible pain). Measurements were performed by the headache specialist during consultation, where a six-item headache impact test (HIT-6) with possible scores from 36-78 was used to assess the impact of headaches on daily life of the patients. In general, a score above 50 is considered high. HIT-6 is validated and reliable for assessing headache impact with a Cronbach alpha of.89-.90 [21]. In our study, the six items associated with HIT-6 scores had good reliabilities (all Cronbach alpha=.84). Two experienced neurologists (Kai Ivar Müller, Svein Ivar Bekkelund) conducted all the consultations. Meanwhile, the telemedicine participants were guided out of sight to the consultant.

To evaluate the feasibility (a-i), cost (j-k), and travel savings (l) of telemedicine in urban and rural patients, the following outcome variables were selected: (a) eligible patients’ acceptance of telemedicine, (b) dropout rates and causes of dropouts (medical or technical reasons), (c) participants’ satisfaction with video quality and sound quality (patients were asked if they were “satisfied” with the video quality and sound quality after each telemedicine consultation; “Yes” or “No, why not?”), (d) technical errors with the telemedicine equipment, and (e) specialist consultation time in minutes. Descriptive variables included (f) diagnostic investigations (CT, MRI, and other), (g) nonpharmacologic advices, (h) number of prescriptions, and (i) follow-up appointments. Cost and travel variables included (j) cost of travel in euros (€) based on the Norwegian Patient Travel Agency probabilistic method [22], (k) estimated loss of income (<3.5 hours=a half day’s salary, >3.5 hours spent on travel and consultation=one day’s salary), (l) traveling distance in kilometers and traveling time in hours as estimated by Google Maps.

The Norwegian Patient Travel Agency calculated the cheapest means of public transport to and from Tromsø University Hospital for every patient. We collected the cheapest cost from the Norwegian Patient Travel Agency from every participating patient in every municipality of our area. Earnings were calculated based on the Norwegian full-time employee’s average salary, which was €4.681 per month in 2014 [23]. We adjusted all costs to the consumer price index (CPI) January 1, 2015 from Statistics Norway and converted Norwegian kroner into euros by using the exchange rate of one Norwegian krone per euro from the Norwegian Bank on December 31, 2014. Since the telemedicine equipment is in use for many different purposes at the University Hospital in Tromsø, we did not include the cost of the equipment, its installation, or its maintenance.

### Statistical Analyses

The computer program SPSS version 21 was used to analyze the data. Continuous variables were tested for normal distribution with the Shapiro-Wilk test, skewness, and kurtosis. Normally distributed variables were presented as mean (SD) and categorical data as numbers and percentages. Independent sample t-tests were used to compare continuous variables and chi-square tests were used to compare categorical variables between groups. Yates’ continuity correction was used for 2 × 2 tables. In addition, skewed variables are presented as median and range, while the Mann-Whitney U test was used to compare continuous variables. All variables were first tested in univariate models. Statistical significance was defined as *P*<.05.

Hierarchical multiple linear regression was used to assess the ability to predict VAS pain scores of patient’s from rural locations when adjusted for other variables. After sex and age, only variables that could be associated with changes in pain scores and that showed significance in the univariate analysis were selected. Hierarchical multiple linear regression was also used to assess the ability to predict the waiting time of patients from rural locations. We adjusted for age, sex, and other predictors. Furthermore, nonparametric variable waiting time was log transformed in the regression model. Normal probability plot (P-P) of the regression standardized residuals, histogram, and scatterplots were used to assure normality, linearity, and residual independence as well as to eliminate outliers in both models. In addition, multicollinearity was checked by tolerance and variance inflation factor (VIF) in both models.

### Consent and Ethical Approval

Oral and written consent from all participants was obtained. The participants’ mental and physical integrity has been fully respected and safeguarded in accordance with the Helsinki Declaration [24]. The study was approved by the Norwegian National Committee for Medical and Health Research Ethics (REC), number 2009/1430/REK.

## Results

All referred nonacute headache patients (N=557) were screened, and 402 of 486 (82.7%) candidates were included in the final analyses (Figure 1). From 409 specialist consultations, 7 (1.7%, 7/409) patients were excluded. Of the eligible individuals, 402/479 (83.9%) accepted telemedicine, 57/479 (11.9%) did not approve telemedicine, and 20/479 (4.2%) declined specialist care. One patient in the traditional consultation group was excluded due to a transient left-sided hemiparesis observed in his headache history. Second patient was excluded as the maximum waiting time was exceeded. Also, in the telemedicine group, one patient was excluded due to a cystic cerebellar lesion seen on an MRI. Another had ataxia, and the third had suspect subcutaneous tumors on his scalp. Additionally, one patient had been seen by a neurologist within a 2-year period prior to the referral, and another patient was excluded because the consulting telemedicine room was occupied.

All included patients underwent only MRI/CT brain scan (205/402 participants, 51.0%), only neurological examination (46/402, 11.4%), or both (151/402, 37.6%). The study participants did not differ significantly from the excluded individuals by sex and age (n=155, *P*=.17 and .41, respectively).

**Figure 2:**
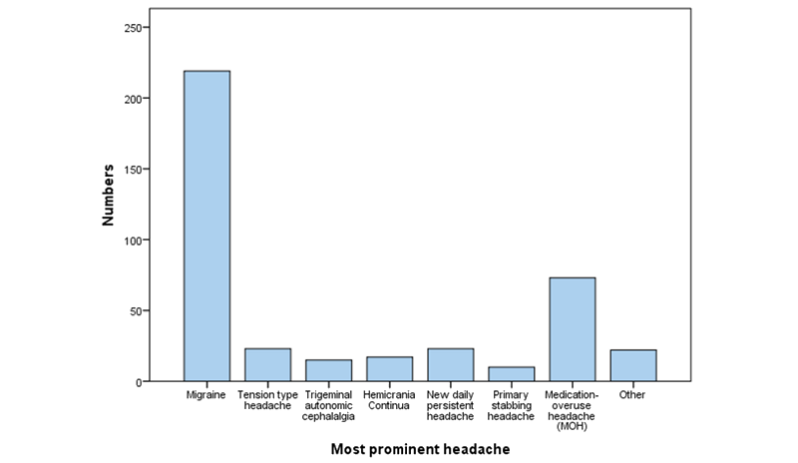
Overview of the most prominent headaches, n=402.

Migraines (219/402 patients, 54.5%) and probable MOH (73/402 patients, 18.2%) were the most common referral reasons (Figure 2). The most frequent comorbid disorders were chronic neck pain (188/402, 46.8%) and insomnia (126/402, 31.3%), followed by hypertension (36/402, 9.0%), asthma (21/402, 5.2%), depression (18/402, 4.5%), and hypothyreosis (15/402, 3.7%). Two participants had a history of cerebrovascular disease, and one had epilepsy. Patients who underwent traditional consultations did not significantly differ statistically by demographics and clinical characteristics as compared to the patients in the telemedicine group (Table 1).

**Table:** Baseline characteristics of patients with headache who were referred to specialists.

Consultation form Characteristic		TelemedicineN=200	Traditional N=202	*P*-value
Females, n (%)		148 (74.0)	153 (75.7)	.77
Married/cohabitating, n (%)		117 (58.5)	135 (67.0)	.09
Age, years, mean (SD)		36.0 (13.0)	38.0 (13.7)	.12
Education, years (SD)		13.5 (3.0)	13.8 (3.1)	.22
Employment full time, n (%)		128 (64.0)	125 (61.9)	.74
Unemployed, n (%)		11 (5.5)	19 (9.4)	.19
Sick leave due to headache, n (%)		58 (29.0)	62 (30.7)	.79
^a^ **Headache days/month, n (%)**				.51
	> 15 days	120	113	
	7-15 days	41	40	
	< 7 days	39	49	
HIT-6, mean (SD)		64.1 (6.1)	64.0 (6.1)	.49
VAS, mean (SD)		7.1 (2.2)	6.9 (2.1)	.82
**Most prominent headache**				
	Migraine, n (%)	106 (53.0)	113 (55.9)	.62
	TTH, n (%)	15 (7.5)	8 (4.0)	.19
	MOH, n (%)	35 (17.5)	38 (18.8)	.83
Waiting time, days, median (range)		63 (117)	59 (119)	.34
Travel cost per patient €, median (range)		83 (437)	83 (437)	.96
^b^Lost pay per patient, €, median (range)		234 (117)	234 (117)	.87

SD: standard deviation; HIT-6: headache impact test-6; VAS: visual analog scale; TTH: tension type headache; MOH: Medication overuse headache.

^a^Last 3 months before headache consultation.

^b^Corresponding to a half day’s salary in the urban area and a day’s salary in rural areas.

### Feasibility Variables

The feasibility variables of the randomized groups are shown in Table 2. Telemedicine had shorter consultations (*P*<.001), especially for men (35.3 vs 43.0 min, *P*=.001). Men had shorter telemedicine consultations than women (35.3 vs 40.0, *P*=.002). Meanwhile, there were no considerable differences in consultation time between men and women in the traditional group (43.0 vs 44.0, *P*=.63). Other feasibility parameters did not differ between the groups (*P*>.05; Table 2). All participants were satisfied with the video quality, and 198/200 (99.0%) were satisfied with the sound quality. In the first case, there was no sound transmission, and the patient had to be consulted through video and the loudspeaker on the ordinary telephone at the office. In the second case, the patient felt the sound was too loud, and the study nurse was called upon to decrease the volume.

A significantly lower share of women were referred from rural areas compared with the urban area (*P*=.04), and the rural women had significantly higher pain scores (*P*=.005). A hierarchical multiple regression model of VAS predictors showed that sex and probable MOH explained 3.5% of the VAS variance (*P*=.003) while the whole model explained 5.1% (*P*=.01). For each rural patient, there was a 0.6 increase on the VAS scale (*P*=.01) when adjusted for age, sex, and diagnosis (Table 3). We did not find other significant VAS predictors than sex, probable MOH, and patient location.

**Table 2:** Clinical and feasibility evaluation of telemedicine consultations in patients who were referred to specialists for headaches.

Consultation form		Telemedicine, N=200	Traditional, N=202	*P*-value
**Clinical variables**				
	Change in diagnosis, n (%)	43 (21.5)	40 (19.8)	.77
	Additional diagnosis, n (%)	109 (54.5)	118 (58.4)	.49
	Additional MRI/CT, n (%)	74 (37.0)	70 (34.7)	.70
	Nonpharmacologic advice, n (%)	162 (81.0)	170 (84.6)	.41
	Prescriptions, n (%)	164 (82.0)	166 (82.2)	1.00
	Follow-up by GP, n (%)	136 (68.0)	129 (63.9)	.44
	Follow-up by neurologist, n (%)	6 (3.0)	6 (3.0)	1.00
**Outcome variables**				
	Dropout, medical reasons, n (%)	2 (1.0)	1 (0.5)	NA
	Dropout, technical failure, n (%)	1 (0.5)	0	NA
	Minor technical issues, n (%)	21 (10.5)	NA	NA
	Satisfied with sound quality, n (%)	198 (99.0)	NA	NA
	Satisfied with video quality, n (%)	200 (100.0)	NA	NA
	Consultation time, min, mean (SD)	38.8 (9.5)	43.7 (12.3)	< .001
	Males, min (SD)	35.3 (8.3)	43 (13.1)	.001
	Females, min (SD)	40.0 (9.6)	44.0 (12.0)	.002
	^a^Preparation to visit, min (range)	14.0 (44)	14.0 (29)	.56

GP: general practitioner; NA: not applicable; SD: standard deviation

^a^The time used by the nurse before each consultation.

**Table 3:** Hierarchical linear regression model to assess rural location as a predictor of VAS when controlled for confounders.

Step		^a^B (standard error)	95% CI	*P*-value
**Step 1**				
	Constant	5.786 (.578)	4.649 to 6.922	<.001
	Age	‑.003 (.008)	‑0.19 to .013	.71
	Sex	.699 (.250)	.207 to 1.191	.01
	MOH	.649 (.279)	.101 to 1.198	.02
**Step 2**				
	Constant	5.220 (.614)	4.013 to 6.427	<.001
	Age	‑.003 (.614)	‑.019 to .013	.69
	Sex	.770 (.250)	.278 to 1.261	.002
	MOH	.637 (.277)	.092 to 1.182	.02
	Urban/rural	.625 (.240)	.153 to 1.096	.01

CI: confidence intervals; MOH: Probable medication overuse headache.

^a^Unstandardized coefficients.

Patients in the rural group had significantly longer waiting times for specialist consultations (median=64 days, range=120) than patients in the urban group (median=47 days, range=112, *P*=.001). A hierarchical multiple linear regression model to assess rural location and predict waiting time after adjusting parameters for age and sex showed a total variance of 4.4% as explained by the model (*P*=.001). Age, sex, marital status, education, employment, sick leave, HIT-6, migraine, tension type headache, and probable MOH were insignificant predictors of waiting time.

### Cost and Travel Estimates

A comparison between urban and rural participants is given in Table 4. The potential of lowest estimated travel cost, loss of pay, and travel burden compared between urban and rural patients is summarized in Table 5. Travel cost per patient was notably higher in rural areas (outside Tromsø city; median=€249.0, range=409) as compared with urban areas (in Tromsø city; €6.0 per patient, *P*<.001).

**Table 4:** Characteristics of headache patients from urban and rural areas who were referred to specialists.

Characteristic		^a^Urban, N=110	^b^Rural, N=292	*P*-value
Females, n (%)		91 (82.7)	210 (71.9)	.04
Married/cohabitating, n (%)		69 (62.7)	183 (72.6)	.31
Age, years, mean (SD)		36.3 (13.0)	37.2 (13.6)	.54
Education, years (SD)		14.0 (3.0)	13.6 (3.0)	.24
Employment full time, n (%)		70 (63.6)	183 (62.7)	.95
Sick leave due to headache, n (%)		28 (25.5)	92 (31.5)	.33
^c^ **Headache days/month, n (%)**				
	> 15 days	62 (56.4)	171 (58.6)	.11
	7-15 days	29 (26.4)	52 (17.8)	NA
	< 7 days	19 (17.3)	69 (23.6)	NA
^d^ **HIT-6, mean (SD)**		63.3 (7.1)	64.4 (5.7)	.15
	Female HIT-6	63.9 (6.8)	64.7 (5.5)	.80
	Male HIT-6	60.3 (7.9)	63.6 (6.0)	.047
^d^ **VAS, mean (SD)**		6.6 (2.4)	7.2 (2.1)	.04
	Female VAS	6.6 (2.4)	7.5 (1.9)	.005
	Male VAS	6.6 (2.3)	6.4 (2.4)	.82
Waiting time, days, median (range)		47 (112)	64 (120)	.001

^a^Urban: patients from Tromsø city.

^b^Rural: patients living outside Tromsø city.

^c^Last 3 months before headache consultation.

^d^HIT-6: headache impact test-6; SD: standard deviation; VAS: visual analog scale.

**Table 5:** Cost and travel burden for headache patients from urban and rural areas who were referred to specialists.

Cost	^a^Urban, N=110	^b^Rural, N=292	*P*-value
Travel cost per patient, €, median (range)	^c^6	249 (409)	<.001
^d^Loss of earnings per patient, euro	117	234	<.001
Total cost per patient, euro	123	483	<.001
Travel, kilometers, median (range)	12 (69)	526 (1892)	<.001
Travel time, hours, median (range)	0.4 (1.2)	7.8 (27.3)	<.001

^a^Urban: patients from Tromsø city.

^b^Rural: patients living outside Tromsø city.

^c^The price of a bus ticket in Tromsø city.

^d^Corresponding to a half day’s salary in the urban area and a day’s salary in rural areas.

## Discussion

### Headache Patients’ Acceptability of Telemedicine

There was high level of acceptance for telemedicine, and only 1% (2/200) was unsatisfied with the technical quality of the consultation in our study. These findings concur with a 2009/2010 survey from a remote area, including 1816 individuals, which reports a social acceptance of telehealth at 77.7% (1356/1745) and a confidence level at 65.8% (1146/1742) [25]. On the other hand, another survey conducted in 2008 with a total of 1634 individuals spread over different European countries shows that 42%-81% (964/1634) of patients with headache are unsatisfied with their health care [9]. Additionally, a similar European survey from 2013 shows that 48 % (929/1935) are unsatisfied with the headache management [9]. The authors concluded that access and availability to health care had previously not been given enough attention when analyzing the burden of headaches [9]. Despite having maintained a strict maximum waiting time of 4 months in our study, patients from rural areas waited significantly longer for headache consultations (Table 4). The potential burden of long and cumbersome traveling together with difficult journey logistics may explain this. In these areas, telemedicine may become a countervailing technology that can remodel access and availability barriers for headaches.

### Telemedicine Feasibility for Nonacute Headaches

Despite the importance of investigating the feasibility of new technology, only case series exist regarding the use of telemedicine for new headache referrals [13,14]. Previous studies had mainly focused on education programs and follow-up treatments [26-28]. We found that the duration of the telemedicine consultations was 5 min (11%, 38.8 vs 43.7 min) shorter than that of traditional consultations. However, there were no statistical differences between telemedicine visits and traditional visits with respect to headache diagnoses, investigations, advices, prescriptions, and follow-up appointments. Telemedicine caused few dropouts. Minor technical errors of sound and video transmissions were quickly dealt with and did not influence the consultations or participants’ satisfaction (Table 2). Almost all patients randomized to telemedicine completed the study without medical or technical problems. Due to no statistical differences of feasibility in favor of traditional consultations, reduced consultation time may also economically favor telemedicine.

Moreover, most women have more home responsibilities than men. Reduced consultation time along with less traveling may therefore also economically favor the patients’ families, especially in rural areas. Additionally, we found that men had telemedicine consultations that were almost 5 min shorter than those of women. This finding may be attributed to the fact that men have a tendency to consult less than women [29]. Nonetheless, we did not find gender differences in the traditional group. Safety and quality of shorter consultations should be evaluated in prospective follow-up studies.

The number of women referred to specialists from rural areas being lower than those from urban areas and rural women having been referred seemingly having higher headache burdens may indicate that rural women consult general practitioners (GPs) for headaches less often than men. This phenomenon may be a consequence of long traveling logistics to see a headache specialist that interferes with women’s working routines and childcare and home responsibilities. However, headache intensity remained significant even after adjustment for sex, employment, education, and age. Different attitudes and referral practices between rural and urban GPs is another explanation, but this idea needs further investigation. It is well known that headaches are underdiagnosed, often misdiagnosed, and suboptimally treated [30-32].

### Cost Savings of Telemedicine Headache Consultations

Among headache patients referred to specialists in Northern Norway, almost three-fourths of them live in rural areas. For each of these patients with headache, our analyses showed that telemedicine consultations may result in a median travel reduction of €249 as well as a saving of €234, corresponding to one day’s work (Table 5).

As we calculated the lowest possible journey expenses per patient, the amount of actual travel costs may be higher. Previous reviews of literature revealed diverging evidence on the cost-effectiveness and patient acceptability of telemedicine [16,33,34]. A cost-consequence study of 2094 patients in a randomized controlled trial found that joint teleconsultations referred by GPs to orthopedists, urologists, ear-nose-throat specialists, gastroenterologists, and other hospital specialists were more expensive by €138 than traditional visits [35]. Even then, the study concluded that total costs for patient travel and loss of pay would be decreased. Meanwhile, in a randomized controlled trial published in 2001, Chua et al found that telemedicine was not cost-effective for neurological outpatient consultations, but none of the patients had headaches [36]. As expected, our study demonstrated that travel costs are highest in rural areas, especially in those without specialist coverage. In Norway, a minimum of traveling expenses is paid by the patients themselves, which may be tax-deductible, but the regional health authority covers most of it and is also responsible for hospital budgets in the region. Reduced travel costs may allow allocation of more money for treatment.

### Advantages and Limitations

A relatively large number of participants, a high response rate, randomization without statistically significant variation in demographics, headache characteristics and the burden between telemedicine and traditional groups (Table 1), and the real-life recruitment design are the main advantages of the this study. Although, organizing teleconsultations inside the hospital provides standardized conditions and promotes internal validity, it is less realistic than local evaluation of the patients. The fact that patients participated in a study, having increased confidence in the treating specialists, may have biased the results. Moreover, the lack of a placebo control group and blinding should not be underestimated; however, designing such a study would be difficult.

### Conclusions

Our study supports telemedicine as a socially accepted, feasible, and cost-saving technology for diagnosing and treating nonacute headaches. A modern interdependent health care system providing simpler, faster, and cheaper services for patients with headache could be implemented. We are obligated to organize and conduct health care systems efficiently, based more on patients’ needs than on the traditional paradigms. However, the quality and safety of employing telemedicine for patients with headache should be assessed in prospective follow-up studies.
